# T‑shaped sling with modified posterior pelvic reconstruction: a technical note with video vignette

**DOI:** 10.20452/wiitm.2025.17983

**Published:** 2025-09-30

**Authors:** Ling Li, Dao ‑Ming Tian, Xing ‑Qi Wang, Ji ‑Hong Shen

**Affiliations:** Pelvic Floor Disorders Diagnosis and Treatment Center, The First Affiliated Hospital of Kunming Medical University, Kunming, Yunnan, China; Department of Urology, The First Affiliated Hospital of Kunming Medical University, Kunming, Yunnan, China

**Keywords:** cystocele, reconstruction, stress urinary incontinence, surgery

## Abstract

To address the lack of standardized procedures for concurrent stress urinary incontinence (SUI), cystocele, and vaginal laxity, we developed a novel, integrated approach that employs a customized inverted T-shaped sling for bladder neck-to-midurethral suspension combined with modified posterior pelvic reconstruction. The widened sling design with posteriorly shifted suspension vector prevents postoperative voiding dysfunction associated with traditional slings. The modified reconstruction technique employs high-strength absorbable barbed sutures for bilateral levator ani plication and perineal body reinforcement, which reduces urogenital hiatus dimensions, corrects vaginal laxity, and prevents long-term recurrence through the enhanced level III support. A 46-year-old woman with concomitant SUI, stage II cystocele (according to the Pelvic Organ Prolapse Quantification system), and vaginal laxity successfully underwent the procedure. Magnetic resonance imaging performed 6 months portsurgery confirmed adequate bladder repositioning. During the 24-month follow-up, SUI symptoms were resolved, pelvic / perineal discomfort diminished, and vaginal laxity during intercourse improved. This technique appears to represent a feasible single-stage solution for women presenting with concurrent SUI, cystocele, and vaginal laxity, providing comprehensive anatomical and functional restoration.

## INTRODUCTION

The prevalence of symptomatic stress urinary incontinence (SUI) is influenced by the anatomical location of pelvic organ prolapse (POP), particularly cystocele.^1^;^2^ reported that 46.3% of patients with SUI presented with cystocele and / or loss of urethrovesical folds, and nearly half of those with cystocele also had concomitant SUI. These patients often experience vaginal laxity and sexual dysfunction.^3^ Over the past 2 decades, the mid-urethral sling (MUS) has been the gold standard treatment for SUI.^4^ However, MUS alone does not correct concomitant cystocele, while conventional antiprolapse surgery restores anatomy but frequently fails to adequately address SUI.^5^;^6^ To overcome these limitations, our team has developed a novel surgical technique: a T-shaped sling with modified posterior pelvic reconstruction.

## AIM

This study aimed to detail the application of this novel surgical technique in a patient with SUI, cystocele, and vaginal laxity. It is supplemented with a video vignette.

## MATERIALS AND METHODS

T-shaped sling with modified posterior pelvic reconstruction has been routinely performed at our center for this patient population. This study describes a 46-year-old woman who presented with involuntary urine leakage during coughing, accompanied by pelvic and perineal discomfort, as well as perceived vaginal laxity during intercourse. The diagnosis involved SUI (urodynamic study showing abdominal leak point pressure of 104 cm H₂O), stage II cystocele (POP quantification system, Aa 0, Ba +1, C –4, gh 4, pb 2.5, tvl 8, Ap –1, Bp -1, D –6), and vaginal laxity (admitting 3 fingers).

During the procedure, nonabsorbable Pelvimesh monofilament polypropylene mesh with ultra-lightweight and large-pore properties (Herniamesh, Chivasso, Italy) was utilized. Prior to implantation, the mesh was tailored into a T-shaped tape (as demonstrated in the supplementary video).

### Surgical technique

The patient was given general anesthesia and placed in the lithotomy position after routine disinfection and draping. A urinary catheter was inserted.

#### Vaginal dissection

**Figure 1:**
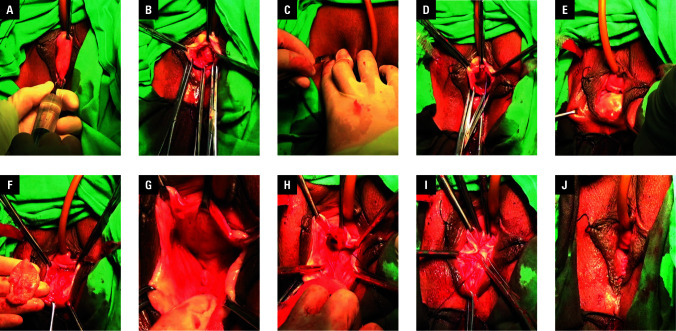
**A** – vesicovaginal hydrodissection; **B** – midline vaginal incision and blunt dissection; **C** – puncture point localization; **D** – flattening and fixation of the tape body; **E** – rectovaginal and perineal hydrodissection; **F** – hexagonal vaginal flap excision; **G** – blunt dissection exposing the levator ani muscles; **H** – horizontal levator plication with barbed sutures; **I** – perineal body reinforcement; **J** – vaginal closure

Hydrodissection with a 0.5:1000 epinephrine / saline solution was performed to minimize bleeding and facilitate tissue separation within the vesicovaginal space (Figure 1A). A midline full-thickness vaginal incision was made, extending from the suburethral region to the supracervical area. The bladder neck was localized by palpating a Foley catheter balloon under traction. Blunt dissection was then carried out to expand the anterior vaginal space toward the posterior pubic arch for tape placement (Figure 1B).

#### Graft tape placement

Puncture sites were identified 0.5 cm lateral to the mid-descending pubic rami using osseous landmarks (Figure 1C). Under transvaginal digital guidance, the tape arms were deployed bilaterally via the outside-in transobturator approach. The body of the tape was positioned from the midurethra to the bladder neck and anchored at its apices with 2–0 absorbable sutures (Figure 1D). The mesh was then buried submucosally using a continuous 3–0 absorbable suture before final closure of the vaginal mucosa.

#### Posterior pelvic reconstruction

Hydrodissection was performed in the rectovaginal space and perineal body (Figure 1E). A hexagonal vaginal flap was excised from the hymenal ring toward the cervical os (Figure 1F). Blunt dissection exposed the bilateral levator ani muscles (Figure 1G). Horizontal levator plication was performed using 2–0 barbed sutures (Ethicon SXPP1A403, Raritan, New Jersey, United States; tensile strength retention, 5 mo), to approximate the muscle bundles and reduce the diameter of the levator hiatus (Figure 1H). Perineal body reinforcement via fascial–muscular approximation thickened and elongated the structure, closing the urogenital hiatus (Figure 1I). The vaginal incision was closed with continuous absorbable sutures (Figure 1J), and iodinated vaginal packing was placed for hemostasis.

### Ethics

This study was approved by the hospital’s Ethics Committee (2024-L-127) and written informed consent (including consent for image and video presentation) was obtained from the patient.

## RESULTS

**Figure 2:**
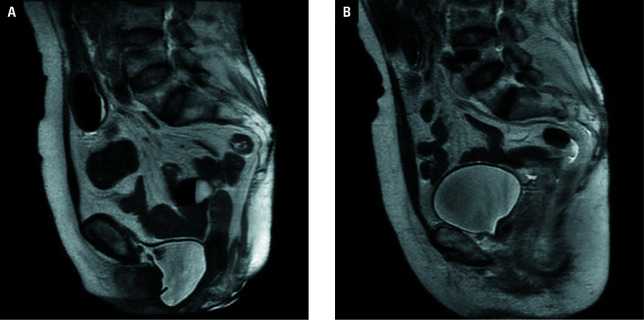
**A** – preoperative pelvic magnetic resonance imaging (MRI; Valsalva maneuver); **B** – 6-month postoperative pelvic MRI (Valsalva maneuver)

The surgical procedure lasted 50 minutes and proceeded uneventfully. The urinary catheter was removed on the second postoperative day, followed by patient discharge. At the 24-month postoperative follow-up, the patient showed satisfactory recovery with complete resolution of SUI symptoms and pelvic discomfort, alongside reported improvement in sexual function. Postoperative magnetic resonance imaging at 6 months confirmed adequate restoration of bladder positioning Figure 2.

## DISCUSSION

For patients with SUI combined with cystocele, urogynecologists typically perform native-tissue anterior colporrhaphy. However, compared with graft-based procedures, native tissue repair is associated with significantly higher long-term recurrence rates, and the overall effectiveness of traditional anterior colporrhaphy remains suboptimal.^7^ For isolated SUI, MUS demonstrates favorable clinical outcomes, with reported 10-year postoperative cure and improvement rates of 63.3% and 80%, respectively.^8^ Nevertheless, in complex cases involving multiple conditions—such as concurrent SUI, cystocele, and vaginal laxity—no standardized treatment protocol currently exists. Surgeons often resort to combined procedures, such as anterior mesh implantation (or anterior colporrhaphy) plus MUS and perineorrhaphy or vaginal tightening. In an effort to address both cystocele and SUI simultaneously, some experts have proposed a modified technique using anterior 4-armed mesh.^9^ Despite this innovation, the approach continues to show limited effectiveness in achieving sustained SUI resolution and does not adequately address concomitant vaginal laxity.

**Figure 3:**
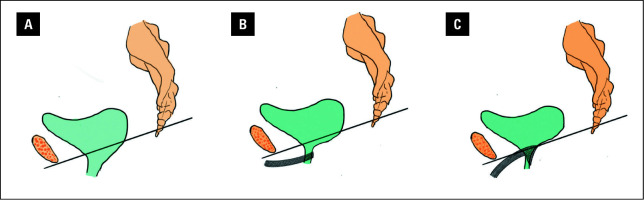
**A** – schematic illustration of concomitant SUI and cystocele; **B** – postoperative midurethral sling placement schematic illustration; **C** – configuration of the implanted T-shaped sling

It is evident that conventional surgical approaches fail to comprehensively address all aspects of complex pelvic floor dysfunction in a single procedure. Moreover, MUS, currently considered the most effective treatment for SUI, carries a risk of voiding dysfunction, with a reported average incidence of 5.53% that closely correlates with surgical experience.^10^ A key advantage of the inverted T-shaped sling technique is eliminating sling tension adjustment, enabling maximal tightening without voiding complications. The widened body of the sling offers extensive support extending from the bladder neck to the midurethra, thereby increasing the area of suspension and shifting its gravitational center posteriorly—from the midurethra toward the bladder neck Figure 3. This design helps prevent urethral obstruction and subsequent voiding dysfunction.

Additional advantages of the technique include: 1) enhanced surgical safety and reduced risk of inguinal pain through puncture site localization using bony landmarks rather than skin markers; 2) reconstruction of the levator ani and perineal body, which narrows the urogenital hiatus, improves vaginal laxity, and reinforces level III support to minimize recurrence^11^; and 3) simultaneous management of rectocele via intraoperative reinforcement of the rectovaginal fascia.

However, the use of nonabsorbable mesh carries a risk of mesh exposure. Reported rates range from 2% to 30%, with most cases manageable through outpatient excision of the exposed segment or conservative treatment.^12^ Mesh exposure is frequently associated with insufficient pore size, imprecise dissection, postoperative bleeding, or infection.^13^ To mitigate this risk, the following measures were implemented in our technique: 1) selection of an ultra-lightweight, large-pore mesh; 2) full-thickness vaginal incision following hydrodissection to preserve vascular integrity; 3) adequate dissection to ensure the mesh was fully flat without folds; 4) embedding the mesh with absorbable sutures to isolate it from the incision site; and 5) emphasizing postoperative vaginal care and regular follow-up for early complication detection. As with traditional MUS and other vaginal procedures, intraoperative risks, such as bladder or rectal injuries, necessitate meticulous anatomical dissection. Thus, this procedure demands that the surgeon possess extensive experience in vaginal surgery.^12^

It should be emphasized that this study represents a single-case demonstration of a novel procedure. Although the 24-month complication-free follow-up is promising, these findings are preliminary. This technical note primarily aims to describe the surgical anatomy and procedural details of this approach. Larger comparative studies with longer follow-up are warranted to validate its safety and efficacy relative to existing treatments.

## CONCLUSIONS

This integrated technique appears to represent a viable single-stage approach for the management of women with concomitant SUI, cystocele, and vaginal laxity, offering both anatomical correction and functional improvement. Future studies with more comprehensive experimental designs are needed to validate the long-term efficacy of this technique.
